# Environmental sustainability in asthma: reducing carbon footprint and medication wastage

**DOI:** 10.1186/s13223-025-00988-x

**Published:** 2025-09-29

**Authors:** Ming Ren Toh, Shu Wei Ang, Gerald Xuan Zhong Ng, Ishita Goel, Kai Xin Low, Vivian Tan, Kheng Yong Ong, Hong Ngee Chan, Jun Tian Wu, Chun Fan Lee, Marcus Eng Hock Ong, David Bruce Matchar, Ngiap Chuan Tan, Chian Min Loo, Shao Wei Lam, Mariko Siyue Koh

**Affiliations:** 1 Department of Respiratory and Critical Care Medicine, Singapore General Hospital, Singapore, Singapore; 2 Duke-NUS Medical School, Singapore, Singapore; 3 School of Physical and Mathematical Sciences, Nanyang Technological University, Singapore, Singapore; 4 Division of Pharmacy, Singapore General Hospital, Singapore, Singapore; 5 Duke-NUS Medical School, Health Services Research, Singapore, Singapore; 6 Department of Emergency Medicine, Singapore General Hospital, Singapore, Singapore; 7 SingHealth Polyclinics, Singapore, Singapore

**Keywords:** Carbon footprint, Asthma inhalers, Medication wastage, Oversupply

## Abstract

**Introduction:**

Asthma inhalers are significant contributors of greenhouse gas emissions. However, less is known about the potentially avoidable carbon footprint i.e. medication wastage and oversupply. We aimed to analyse dispensing patterns and carbon footprints of asthma inhalers, quantify medication wastage, and identify determinants of medication oversupply.

**Methods:**

We reviewed the asthma-related dispensation records from 2015 to 2019, in an anonymised, cluster-wide repository linking electronic medical, pharmacy and administrative records, containing patient and visit details on demographics, comorbidities, GINA step, and site of care. Medication wastage, a visit-level measure, was defined as the number of inhalers dispensed in excess of the quantity required during each refill interval. Medication oversupply, a patient-level aggregated measure defined by medication possession ratio (MPR) > 1.2, where MPR equals total dispensed days (summed across all maintenance inhalers) divided by the follow-up period. All analyses were performed using R Studio.

**Results:**

205,337 inhaler units were dispensed over the study period, contributing an estimated 1,541,591 kgCO2e. The most frequently prescribed inhalers were SABA MDIs (79,007 units; 38.5%), followed by ICS-LABA MDIs (46,335 units; 22.6%), ICS MDIs (36,635 units; 17.8%), ICS-LABA DPIs (33,730 units; 16.4%), and ICS DPIs (9,630 units; 4.7%). ICS-LABA MDIs remained the greatest contributor of carbon footprint, with annual carbon emissions nearly doubling from 114,476 kgCO2e in 2015 to 214,575 kgCO2e in 2019. A total of 6,427 canisters were dispensed in excess of refill intervals, accounting for 46,798 kgCO2e. Beclomethasone MDIs accounted for the majority of wasted inhalers. In a multinomial regression analysis, patients receiving care in primary care settings were significantly more likely to be oversupplied medications compared to those in specialist care (OR 1.93, 95% CI 1.49–2.51).

**Conclusion:**

ICS-LABA MDIs are the predominant source of inhaler-related carbon footprint, with additional contribution from excessive dispensation of inhalers.

**Electronic supplementary material:**

The online version of this article (10.1186/s13223-025-00988-x) contains supplementary material, which is available to authorized users.

## Introduction

Asthma affects over 260 million people worldwide, and metered dose inhalers (MDIs) contribute approximately 0.03% of global greenhouse gas emissions [1]. Since the implementation of the Montreal Protocol, there has been a growing focus on sustainable asthma care [2]. For instance, the National Health Service (NHS) has pledged to be carbon neutral by 2045, and the Global Initiative for Asthma (GINA) has repeatedly emphasized the importance of environmental sustainability [1].

Inhaled therapies, such as metered dose inhalers (MDIs), are critical for asthma management but contribute significantly to healthcare’s carbon footprint especially during the use and disposal phases of its life cycle [3]. Hydrofluorocarbons (HFCs), commonly used as propellants in pressurized MDIs, were responsible for 18 million tonnes of carbon dioxide equivalent (CO2e) emissions globally in 2018 [1, 4]. In the United Kingdom, 70% of all inhalers are MDIs, which contribute 13% of the National Health Service's (NHS) total carbon emissions [1, 5]. In contrast, dry powdered inhalers (DPIs) produce over 95% less carbon emissions than MDIs [1]. Nevertheless, DPIs can also negatively impact the environment beyond inhaler-related CO2 emissions, contributing to issues like soil acidification and freshwater eutrophication.[1, 18] Beyond inhaler-related CO2 emissions, medication wastage poses a major, understudied barrier in sustainable asthma care. Patients are often dispensed multiple-dose relievers for short-term use during exacerbations, and prescribers often prescribe quantities in excess of their needs before the next refill. A single center in the United States reported that 87% of dispensed inhaler doses were unused and discarded, with a projected cost exceeding USD 230 million [6]. Similarly, inhaled corticosteroid (ICS) oversupply ranges from 12 to 29% in Swedish pharmacy databases, 27% in a cohort of 44 general practitioners from NHS Forth Valley, Scotland, and 11–34% in a hospital in Thailand [7–9] .

Amidst the growing awareness of inhaler propellants impact on global CO2 emissions and medication wastage, less is known about the carbon footprint of wasted inhalers. To this end, we will review the dispensation trends and carbon footprints of all asthma inhalers, quantify the extent of medication wastage and its associated CO₂ emissions, and identify factors linked to medication oversupply.

## Methods

### Study setting and participants

We performed a retrospective, observational study of adult asthma patients (aged ≥ 18 years old) managed within Singapore’s largest public healthcare network from 1 January 2015 to 31 December 2019. Our healthcare cluster comprised primary care in nine community polyclinics, as well as secondary care in the respiratory specialist clinics and tertiary care hospital, and covers about 20% of Singapore’s adult population [10]. Ethics approval was obtained from the SingHealth Centralised Institutional Review Board (2017/2950).

### Eligibility criteria

Asthma diagnosis was identified using the International Classification of Diseases (ICD) codes for asthma, including ICD-9 code 493 and ICD-10 codes J45-J46.

### Data sources and variables

Data were sourced from the SingHealth Chronic Obstructive Pulmonary Disease (COPD) and Asthma Data Mart (SCDM), a comprehensive real-world database with integrated data from electronic medical, pharmacy and administrative records within the healthcare cluster [11]. The anonymised dataset contains patient-level and visit-level information on demographics (age, sex, ethnicity), comorbidities, asthma severity classified by 2015 Global Initiative for Asthma (GINA) steps, site of care (primary, specialist care or shared care involving both sites) and oral corticosteroid (OCS) use [10].

The primary exposure variable was inhaler usage, measured by the number of inhalers dispensed over the five-year study period. For each inhaler, the carbon footprint data (in kilograms of carbon dioxide equivalent, kgCO2e) based on the inhaler propellant were summarised in Supplemental Table 1. Medication wastage for each visit (*x*), expressed in canisters, was calculated as: $$ \begin{aligned} & {\text{Excess}}_{x} = {\text{Dispensed}}_{x} \\ & \quad - \left\lceil {\frac{{{\text{Refill}}\;{\text{interval}}\; \times \;{\text{Prescribed}}\;{\text{daily}}\;{\text{dose}}}}{{{\text{Doses}}\;{\text{per}}\;{\text{canister}}}}} \right\rceil \\ \end{aligned} $$ where ⌈ ⌉ denotes the required number of canisters is rounded up to the next whole canister to avoid under-prescribing. Patients with a single visit during the study period were excluded from the calculation.

For each patient, the medication possession ratio (MPR) was calculated as the total number of dispensed days, summed across all maintenance inhalers, divided by the follow-up period. An MPR > 1.2 indicated medication oversupply which reflects potential medication wastage and contributes to avoidable carbon emissions across the inhalers’ life cycle (including manufacture, transport, use and disposal).

### Data cleaning and handling of missing data

Data cleaning was performed to ensure the quality and reliability of the dataset. This process involved the identification and removal of duplicate records, correcting inconsistencies in variable coding, and addressing missing data. Patients with missing data on inhaler dispensation were excluded from the analysis. We employed complete case analysis for the remaining dataset.

### Statistical analysis

Trends in prescriptions, carbon footprint (kgCO2e), and wastage (in canisters) were evaluated for each inhaler type over 2015–2019. We conducted multinomial regression analysis to assess the associations between patient demographics (age, sex, ethnicity), comorbidities, asthma severity (GINA step), site of care and medication oversupply (MPR > 1.2). Results were presented as odds ratios (ORs) with 95% confidence intervals (CIs). *P*-values < 0.05 denoted statistical significance. All analyses were conducted using R Studio, version 2022.02.2 [12].

## Results

We reviewed the dispensing records across 8,023 adult asthma patients, with a median follow-up of 1,037 days. These included 205,337 canisters, responsible for over 1.5 million kgCO2e emissions (Table 1). The most frequently dispensed inhalers were SABA MDIs (79,007 units; 38.5%), followed by ICS-LABA MDIs (46,335 units; 22.6%), ICS MDIs (36,635 units; 17.8%), ICS-LABA DPIs (33,730 units; 16.4%), and ICS DPIs (9,630 units; 4.7%) (Table 1).

**Fig. 1:**
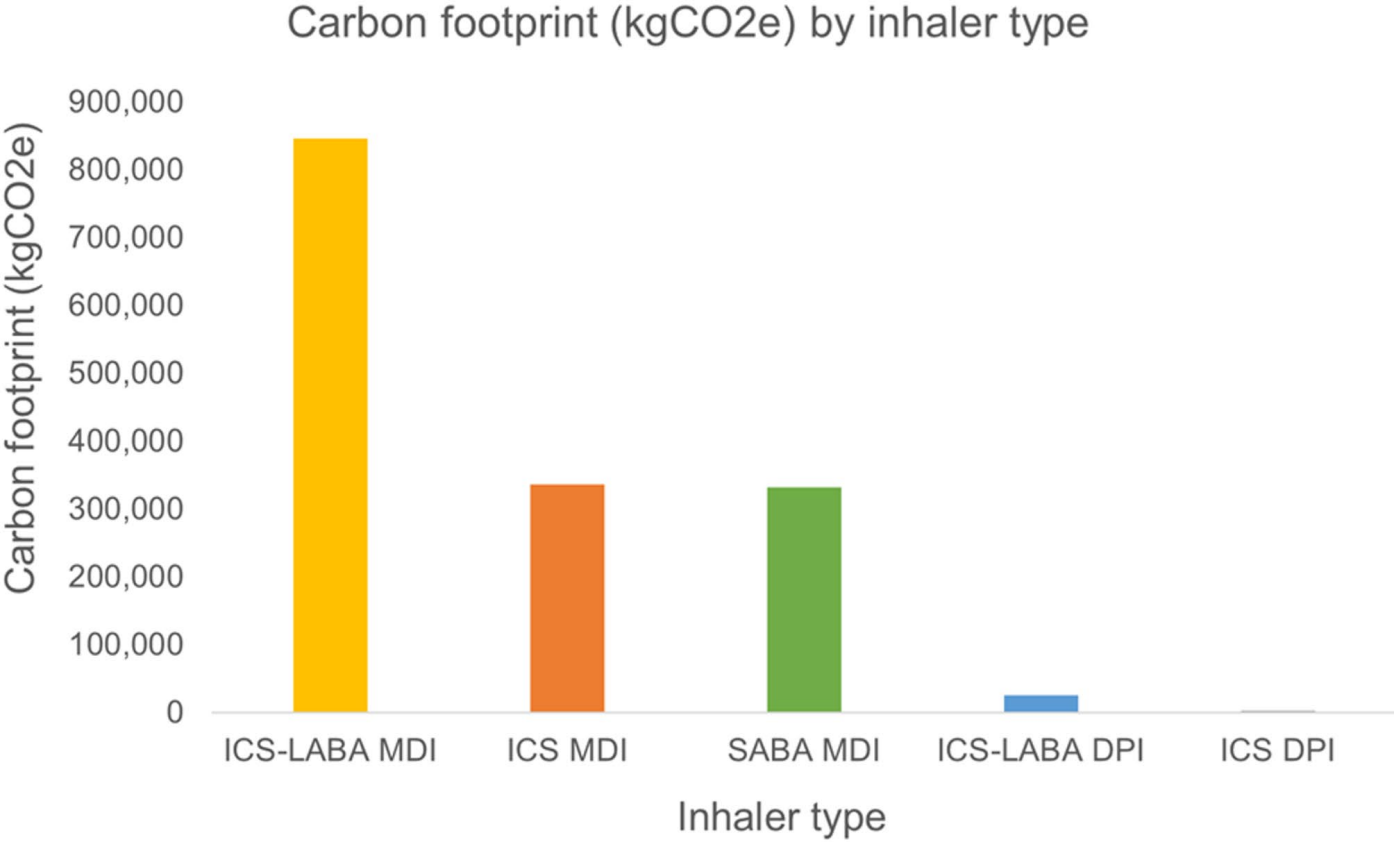
Distribution of carbon footprint (kgCO2e) by inhaler type across the study period. The total carbon emissions from inhaler use were primarily driven by ICS-LABA MDIs. The contribution of DPIs was minimal compared to MDIs. Abbreviations: ICS (inhaled corticosteroids), ICS-LABA (inhaled corticosteroid-long-acting beta-2 agonist), DPI (dry powdered inhaler), MDI (metered dose inhaler), SABA (short-acting beta-2 agonist)

ICS-LABA MDIs generated over half of all emissions (846,102 kgCO2e). ICS MDIs and SABA MDIs each contributed about one-fifth (ICS MDI: 336,146 kgCO2e and SABA MDIs: 331,829 kgCO2e) (Fig. 1). Dry powder inhaler (DPI) formulations accounted for less than 3% of the carbon footprint (28,514 kgCO2e) (Table 1).

**Table 1:** Temporal trends in the carbon footprint and usage of the asthma inhalers

	Number of canisters dispensed (carbon footprint, in kgCO2e)					
Year	ICS MDI	ICS DPI	ICS-LABA MDI	ICS-LABA DPI	SABA MDI	Total
2015	7,603 (68,143)	2,269 (740)	6,270 (114,476)	4,503 (3,377)	14,256 (59,875)	34,901 (246,611)
2016	7,627 (68,641)	2,222 (705)	8,281 (151,229)	6,167 (4,630)	15,551 (65,314)	39,848 (290,519)
2017	7,478 (68,406)	1,918 (634)	9,445 (172,433)	7,030 (5,278)	16,651 (69,934)	42,522 (316,685)
2018	7,074 (66,333)	1,707 (603)	10,593 (193,389)	7,438 (5,591)	16,613 (69,775)	43,425 (335,691)
2019	6,853 (64,623)	1,514 (503)	11,746 (214,575)	8,592 (6,453)	15,936 (66,931)	44,641 (353,085)
2015–2019	36,635 (335,146)	9,630 (3,185)	46,335 (846,102)	33,730 (25,329)	79,007 (331,829)	205,337 (1,541,591)

**Fig. 2:**
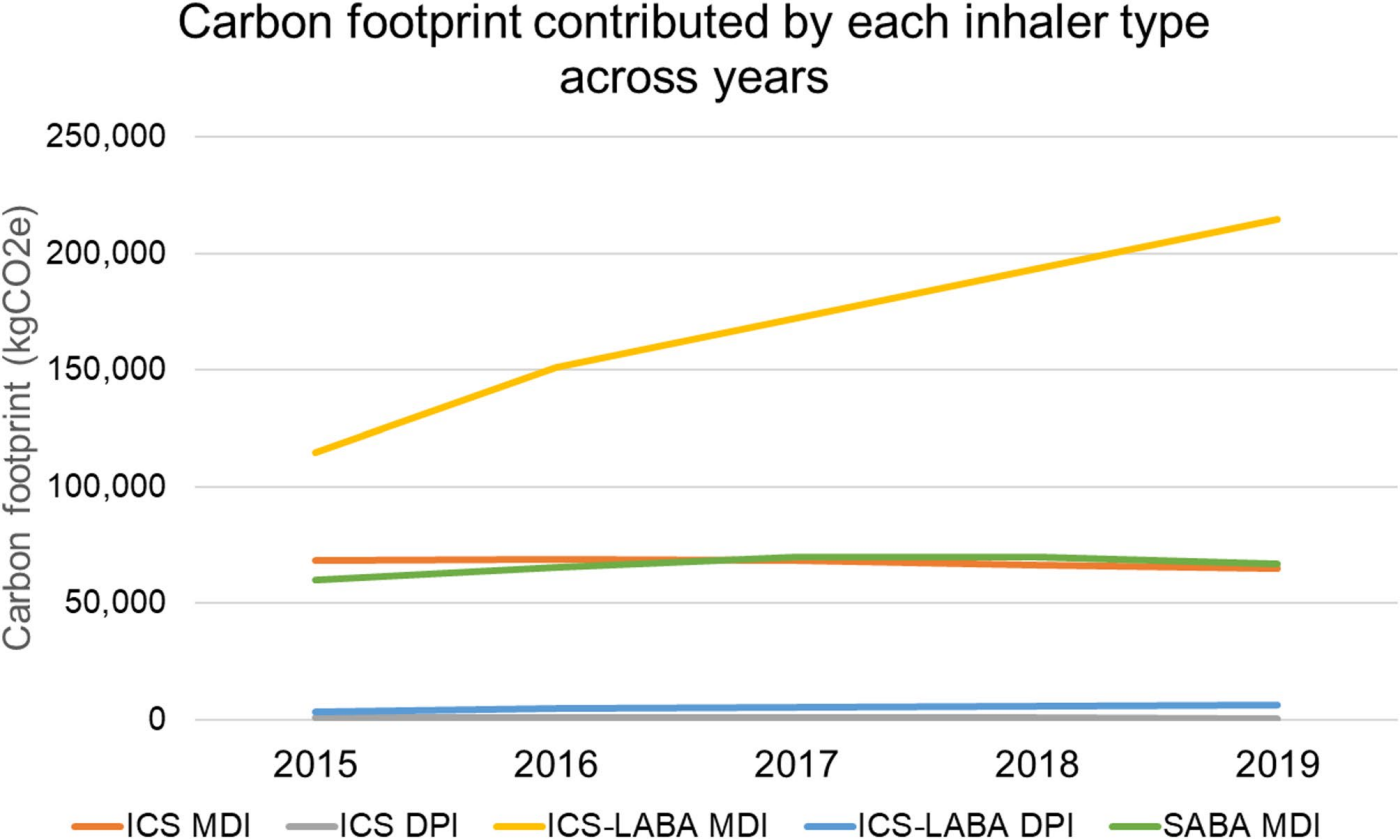
Temporal trends in the carbon footprint (kgCO2e) contributed by each inhaler type from 2015 to 2019. Carbon emissions from ICS-LABA MDIs increased steadily over the five-year period and remained the main contributor throughout. ICS MDIs and SABA MDIs showed relatively stable contributions, while DPIs (both ICS and ICS-LABA formulations) accounted for a consistently small share of emissions. Abbreviations: ICS (inhaled corticosteroids), ICS-LABA (inhaled corticosteroid-long-acting beta-2 agonist), DPI (dry powdered inhaler), MDI (metered dose inhaler), SABA (short-acting beta-2 agonist)

Annual emissions from ICS-LABA MDIs almost doubled from 114,476 kgCO2e in 2015 to 214,575 kgCO2e in 2019, while emissions from SABA MDIs remained stable at 59,875–69,775 kgCO2e (Fig. 2). Of the ICS-LABA MDIs, budesonide/formoterol MDIs had the highest carbon footprint, almost 100 times greater than their DPI counterparts (31.2–64.2 versus 0.75 kgCO2e per canister) (Supplemental Table 1).

To better assess excess dispensation, we analysed medication wastage, defined as canisters dispensed beyond recommended refill intervals. In total, 6,427 canisters were classified as excess, contributing to 46,798 kgCO2e in avoidable emissions. Most of the carbon footprint originated from wasted beclomethasone dipropionate MDIs (4,686 canisters; 40,300 kgCO2e) (Table 2). This analysis reflects the carbon footprint of wasted canisters only.

**Table 2:** Medication wastage, number of canisters and carbon footprint of each inhaler type

Inhaler (type/strength)	kgCO2e/can	Excess canisters	Excess kgCO2e
Beclomethasone dipropionate 250mcg MDI	8.6	2,833	24,363.8
Beclomethasone dipropionate 50mcg MDI	8.6	1,853	15,935.8
Budesonide 100mcg Easyhaler DPI	0.182	2	0.4
Budesonide 200mcg Easyhaler DPI	0.182	1,032	187.8
Budesonide 200mcg Turbuhaler	0.75	366	274.5
Fluticasone propionate 125mcg Evohaler MDI	17.7	281	4,973.7
Fluticasone propionate 50mcg Evohaler MDI	17.7	60	1062.0
All inhalers	–	6,427	46,798

Only inhalers / formulations with wastage were included in this table

**Fig. 3:**
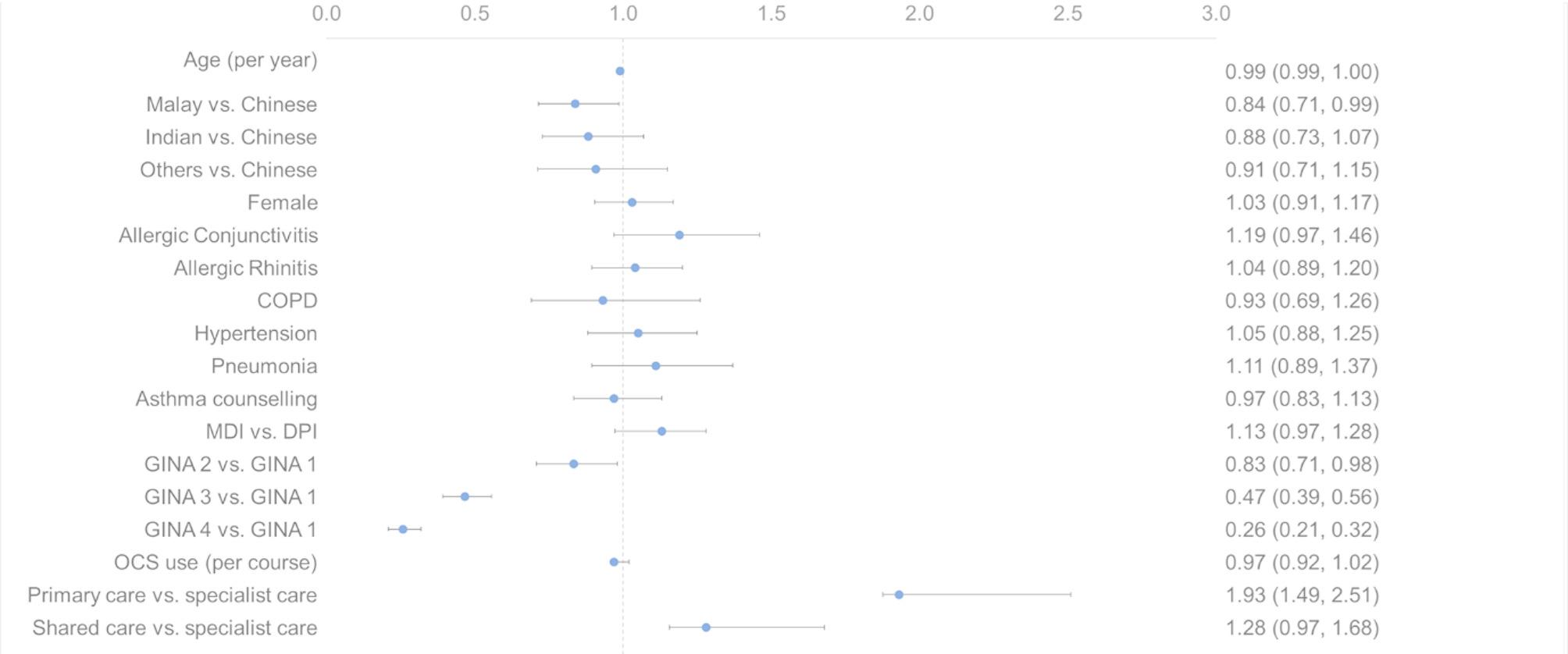
Factors associated with medication oversupply, displayed as a forest plot of odds ratios with 95% confidence intervals. GINA step 5 was excluded from the forest plot as no patients in this group met the criteria for medication oversupply (MPR > 1.2), rendering the odds ratio non-estimable. Abbreviations: CI (confidence interval), COPD (chronic obstructive pulmonary disease), MDI (metered dose inhaler vs. dry powdered inhaler)

Separately, we conducted a patient-level analysis of medication oversupply, defined by a medication possession ratio (MPR) > 1.2 over the follow-up period. This identified 1,956 patients (24.4%) as having persistent oversupply. In the multinomial regression analysis, medication oversupply was more common among patients managed in primary-care settings than among those in specialist care (OR 1.93, 95% CI 1.49, 2.51) (Fig. 3). Patients on higher GINA treatment steps were less likely to be oversupplied compared to those on GINA step 1 (GINA 2: OR 0.833; GINA 3: OR 0.467; GINA 4 OR 0.257) (Fig. 3).

## Discussion

ICS-LABA MDIs contributed the largest share of inhaler-related carbon emissions, a trend likely to continue. This is partly due to increased volumes dispensed following the GINA 2019 recommendations, which advocate for earlier use of anti-inflammatory relievers and single-maintenance-and-reliever-therapy (SMART) [13]. Additionally, ICS-LABA MDIs such as Seretide and Symbicort have a higher global warming potential (GWP) compared to other inhaler types, further amplifying their contribution to the total carbon footprint. Both MDIs contain HFCs with the highest GWP: HFC 227a (3,350 GWP) and HFC 134a (1,300 GWP).[4] Similarly, mometasone/formoterol MDIs were found to have one of the highest per-canister carbon footprints (48.1 kgCO2e), according to an analysis of Medicare and Medicaid claims data, which collectively account for approximately 40% of retail prescription drug spending in the United States [14].

To reduce the carbon footprint, prescribers could consider switching suitable patients from ICS-LABA MDIs to DPIs, which can achieve a reduction in emissions over 90% [1, 15]. A post-hoc analysis of the Salford Lung Study in asthma reported that switching from MDIs to DPIs resulted in a greater than 50% reduction in carbon emissions without compromising asthma control [16]. A separate modelling study projected an annual MDI-to-DPI switch rate of 2–5% could achieve a 38–58% reduction in inhaler-related emissions and a 2–4% reduction in the social cost of carbon [17]. Notably, the Salford Lung Study also found that once-daily DPI regimens improved adherence, potentially offering better asthma control compared to twice-daily MDI regimens [16, 17].

However, switching inhalers may not always be feasible. Poor inhaler technique with DPIs has been observed in 34–44% of patients, compared to 12% with MDIs, posing particular challenges for the elderly and those with reduced inspiratory flow [18–20]. The consequences of improper DPI use can be costly, an estimated £782 million in healthcare costs and productivity losses due to poor DPI technique has been documented across Spain, Sweden, and the UK [20]. Therefore, it is important to minimise carbon footprint via other means, including reducing medication wastage.

Medication wastage in asthma has been described in the context of premature switching of maintenance therapies or the accumulation of partly used rescue inhalers during acute exacerbations [21, 22]. However, less is known about wastage resulting from excess dispensation. We reported an excess of 6,427 canisters of maintenance inhalers dispensed beyond the refill duration, wastage which could be avoidable with judicious prescribing. The true burden of medication wastage is likely greater than our estimates, including wastage from frequent regimen switches and excessive SABA dispensed. This highlights the need to address medication oversupply, a related but broader issue contributing to both wastage and avoidable carbon emissions.

Medication oversupply was common in our cohort (24.4%), especially among those seen in the primary care setting, consistent with previous studies reporting rates between 12 and 34% [7–9]. We found that oversupply was less frequent among patients on higher GINA steps or under specialist care, possibly due to the closer monitoring by multidisciplinary teams. Beyond patient factors, healthcare system factors may also influence medication oversupply rates. In a Swedish study, patients with full payment exemptions had higher rates of medication oversupply compared to those on copayment system (33% versus 19%) [9]. In Singapore, where the public healthcare system operates on a co-payment model, it would be relevant to explore whether financial subsidies influence medication oversupply [23, 24].

Responsible and patient-centred prescribing is crucial to avoid unnecessary inhaler switches and excessive dispensation.[25] Notably, educational initiatives on green prescribing in emergency settings have achieved a 43% reduction in MDI dispensations.[26] Other strategies to reduce medication wastage include routine assessment of inhaler technique prior to refills and early identification of adherence barriers, which can prevent the unnecessary dispensing of inhalers that may otherwise go unused [25]. Pharmacists and asthma coordinators play a critical role by educating patients on disease management, correct inhaler technique, and the environmental consequences of medication waste [25]. Some healthcare systems have even implemented inhaler donations and recycling as part of broader sustainability efforts [25]. We have summarised several steps to reduce carbon emissions in asthma care under Supplemental Table 2.

### Study strengths and limitations

Globally, few studies have quantified the overall carbon footprint of asthma inhalers beyond SABA use [27]. One multicountry study involving 28 countries (using IQVIA MIDAS and SABINA III data) primarily recruited patients from specialist care, limiting the generalizability of its findings to the broader asthma population.[27] The Singapore arm of SABINA III, for example, included only 205 patients, recruited through purposive sampling, with data manually entered into case report forms—introducing potential selection, recall, and transcription biases [28]. In contrast, we reviewed data from more than 70% patients managed in primary care, which is more representative of real-world prescribing practices. Data were also extracted directly from electronic medical records, reducing the risk of data entry error and recall bias, and enhancing both scale and validity. Another strength of our analysis is the direct quantification of medication wastage based on refill intervals, an aspect that is unaddressed in previous carbon footprint studies. This allowed us to identify avoidable emissions from excess dispensation.

However, our study findings are limited by an underestimation of the actual carbon footprint in asthma care. First, we did not account for emissions beyond inhaler use. We were also unable to quantify the number of discarded doses in used canisters. Inhaler manufacturing, and disposal via landfill or incineration can contribute additional CO₂ emissions beyond the use phase.[29] Second, we did not include long-acting muscarinic antagonist (LAMA) inhalers and single-inhaler triple therapies which were primarily used in chronic obstructive pulmonary disease at the time of study recruitment. Third, our estimates did not include emissions from other components of healthcare utilisation, such as clinic operations or patient commute, which also contribute to the overall carbon footprint in asthma care [30].

## Conclusion

Excessive use of MDIs and medication wastage are significant contributors to asthma-related carbon emissions. As climate change continues to accelerate, there is an urgent need to raise environmental awareness in healthcare and implement best practices that balance sustainability and optimal asthma care.

## Electronic supplementary material

Below is the link to the electronic supplementary material.

Supplementary file 1

## Data Availability

The datasets used and/or analysed during the current study are available from the corresponding author on reasonable request with de-identified patient data subject to data owners and other stakeholders’ approval. References for Supplemental Tables 1 & 2 are provided here [31–33].
