# Supplementary material for: Environmental sustainability in asthma: reducing carbon footprint and medication wastage
Source: Allergy Asthma Clin Immunol. 2025 Sep 29;21:42. doi: 10.1186/s13223-025-00988-x (PMC12482071; doi:10.1186/s13223-025-00988-x)
Supplement: Supplementary file 1 [file 13223_2025_988_MOESM1_ESM.docx]

Supplemental table 1. Carbon footprint of the asthma inhalers.

| Formulation | Brand and Strength | Gases; Components | *kg/CO_2_e (per canister) | Strength correlate with the publication | References |
| --- | --- | --- | --- | --- | --- |
| DPI | Anoro Ellipta 30D  Umeclidinium/vilanterol 62.5/25mcg | Lactose, magnesium stearate | 0.8 | Low CO_2_ | [29] |
| DPI | DuoResp Spiromax 120D  Budesonide/formoterol 160/4.5mcg | Lactose | 0.38 | Low CO_2_ | [31] |
| DPI | Fostair Nexthaler 120D  Beclomethasone/ formoterol 100/6mcg | Lactose, magnesium stearate | 0.92 | Low CO_2_ | [29] |
| DPI | Relvar Ellipta 30D  Fluticasone/vilanterol 100/25mcg | Lactose, magnesium stearate | 0.8 | Low CO_2_ | [29] |
| DPI | Seretide Accuhaler 60D  Fluticasone/salmeterol 50/100mcg | Lactose | 0.75 | Low CO_2_ | [29] |
| DPI | Seretide Accuhaler 60D Fluticasone/salmeterol  50/250mcg | Lactose | 0.75 | Low CO_2_ | [29] |
| DPI | Seretide Accuhaler 60D  Fluticasone/salmeterol 50/500mcg | Lactose | 0.75 | Low CO_2_ | [29] |
| DPI | Symbicort Turbohaler 120D Budesonide/Formoterol 160/4.5 | Lactose | 0.75 | Low CO_2_ | [31] |
| DPI | Incruse Ellipta 30D  Umeclidinium 62.5mcg | Lactose, magnesium stearate | 0.8 | Low CO_2_ | [29] |
| SMI | Spioitlo Respimat 60D  Tiotropium/olodaterol 2.5/2.5mcg | Water for injection, edetate disodium, benzalkonium chloride and hydrochloric acid | 0.22 | Low CO_2_ | [29] |
| SMI | Spiriva Respimat 60D  Tiotropium 2.5mcg | Water for injection, edetate disodium, benzalkonium chloride and hydrochloric acid | 0.22 | Low CO_2_ | [29] |
| MDI | Beclo-asthma 200D  Beclometasone Dipropionate 50mcg | HFA-134a (up to 1mL) | 8.6 | High CO_2_ | [31] |
| MDI | Berodual N 200D  Fenoterol, Ipratropium | HFA-134a (qty not specified), citric acid anhydrous, purified water, ethanol, nitrogen (inert gas) | 6.1 | High CO_2_ | [31] |
| MDI | Flixotide Evohaler 120D  Fluticasone Propionate 50mcg | HFA-134a (qty not specified), lactose | 17.7 | High CO_2_ | [31] |
| MDI | Flixotide Evohaler 120D  Fluticasone Propionate 125mcg | HFA-134a (qty not specified), lactose | 17.7 | High CO_2_ | [31] |
| MDI | Flutiform 120D  Fluticasone, formoterol 250/10mcg | HFA-227, Sodium cromoglicate, Ethanol | 36.5 | High CO_2_ | [29] |
| MDI | Iprovent 200D  Ipratropium Br 20mcg | HFA-134a, anhydrous citric acid, purified water, ethanol | 6.1 | High CO_2_ | [31] |
| MDI | Salbuair 200D  Salbutamol 100mcg | HFA-134a (up to 1mL), lactose | 4.2 | High CO_2_ | [31] |
| MDI | Seretide Evohaler 120D  Salmeterol/ fluticasone propionate 25/125mcg | HFA-134a (qty not specified), lactose | 18.1 | High CO_2_ | [31] |
| MDI | Seretide Evohaler 120D  Salmeterol/ fluticasone propionate 25/250mcg | HFA-134a (qty not specified), lactose | 18.55 | High CO_2_ | [31] |
| MDI | Seretide Evohaler 120D  Salmeterol/ fluticasone propionate 25/50mcg | HFA-134a (qty not specified), lactose | 18.1 | High CO_2_ | [31] |
| MDI | Symbicort Rapihaler 120D  Budesonide/formoterol 80/2.25mcg | HFA-227 (qty not specified), povidone K25, macrogol (polyethylene glycol) 1000 | 64.2 | High CO_2_ | [31] |
| MDI | Symbicort Rapihaler 120D  Budesonide/formoterol 160/4.5mcg | HFA-227 (qty not specified), povidone (polyvinylpyrolidoneK25), macrogol (polyethylene glycol) 1000 | 32.1 | High CO_2_ | [31] |
| MDI | Trimbow 120D  Beclomethasone/formoterol/glycopyrronium 100/6/12.5mcg | HFA-134a, Ethanol, Hydrochloric acid | 13.3 | High CO_2_ | [31] |

Abbreviations used: MDI (metered dose inhaler), DPI (dry powdered inhaler), SMI (soft mist inhaler).

Supplemental table 2. Strategies to reduce carbon emissions in asthma care

| Level | Strategy | Description |
| --- | --- | --- |
| Clinician | Prescribe DPI over MDI where clinically appropriate | DPIs have significantly lower carbon footprint; consider for patients who can generate adequate inspiratory flow |
|  | Step down therapy when appropriate | Avoid overtreatment; follow GINA step-down principles to minimize unnecessary inhaler use and limited benefit from continued high-dose ICS [32] |
|  | Use ICS-formoterol as both reliever and preventer (SMART) | Reduces total inhaler burden and SABA overuse, with better asthma outcomes than SABA PRN |
|  | Reduce and limit SABA supply at each visit | SABA overuse has been associated with poorer asthma control and outcomes [33] |
| Patient | Educate on correct inhaler technique and adherence | Prevents waste and overuse due to poor technique or misunderstanding |
|  | Proper disposal and recycling of used inhalers | Encourages sustainable disposal and recovery of materials |
| Institution | Weigh carbon footprint in formulary decision-making | Prefer low-carbon options in procurement policies |
|  | Track and report inhaler prescribing trends | Monitor high-carbon prescribing and provide feedback to prescribers |
| System | Provide DPI options in subsidy lists / essential drug lists | Improve access to low-carbon inhalers through policy |
|  | Develop national guidance integrating clinical and environmental goals | Align sustainability with guideline-based care |

Supplemental table 3. Patient characteristics

| Variable |  | Number of patients (%) |
| --- | --- | --- |
| Total number of patients |  | 8,023 |
| Mean age, years (± SD) |  | 57.05 ± 18.05 |
| Median study follow-up (days) |  | 1,037 |
| Race (%) | Chinese | 4,459 (55.6%) |
|  | Malay | 1,807 (22.5%) |
|  | Indian | 1,120 (14.0%) |
|  | Others | 637 (7.9%) |
| Sex | Male | 3,372 (42.0%) |
|  | Female | 4,651 (58.0%) |
| Multiple (≥3) comorbidities | Yes | 857 (10.7%) |
|  | No | 7,166 (89.3%) |
| Comorbidities | Allergic conjunctivitis | 874 (10.9%) |
|  | Allergic rhinitis | 3,221 (40.1%) |
|  | Anxiety disorder | 66 (0.8%) |
|  | Atopic dermatitis | 12 (0.1%) |
|  | COPD | 501 (6.2%) |
|  | Depressive disorder | 147 (1.8%) |
|  | GERD | 807 (10.1%) |
|  | Heart failure | 327 (4.1%) |
|  | Hypertension | 5,112 (63.7%) |
|  | Obstructive sleep apnoea | 215 (2.7%) |
|  | Pneumonia | 959 (12.0%) |
| Site of care | SC | 1,061 (13.2%) |
|  | PC | 5,647 (70.4%) |
|  | Both SC and PC | 1,315 (16.4%) |
| GINA step at first visit | 1 | 1,753 (21.8%) |
|  | 2 | 2,327 (29.0%) |
|  | 3 | 2,082 (26.0%) |
|  | 4 | 1,858 (23.2%) |
|  | 5 | 3 (<0.1%) |
| ICS inhaler type prescribed at first visit | Metered dose inhaler | 5,552 (69.2%) |
|  | Dry powder inhaler | 2,471 (30.8%) |
| Number of PC visits | 2015 | 13,459 |
|  | 2016 | 14,837 |
|  | 2017 | 15,919 |
|  | 2018 | 17,072 |
|  | 2019 | 17,449 |
| Number of SC visits | 2015 | 46 |
|  | 2016 | 664 |
|  | 2017 | 373 |
|  | 2018 | 511 |
|  | 2019 | 624 |

Abbreviations: COPD (chronic obstructive pulmonary disease), GERD (gastroesophageal reflux disorder), ICS (inhaled corticosteroids), PC (primary care), SD (standard deviation), SC (specialist care).

Supplemental table 4. Proportion of patients with medication oversupply, by site of care (primary care, specialist care, shared care).

|  |  | ICS-LABA | | | ICS | | |
| --- | --- | --- | --- | --- | --- | --- | --- |
|  |  | Number of patients | Proportion of cohort (%) | | Number of patients | Proportion of cohort (%) | |
|  | Year |  | MPR ≤1.2 | MPR >1.2 |  | MPR ≤1.2 | MPR >1.2 |
| All sites | 2015 | 1781 | 76.7 | 23.3 | 2079 | 44.7 | 55.3 |
|  | 2016 | 2324 | 76.3 | 23.7 | 2129 | 42.7 | 57.3 |
|  | 2017 | 2617 | 74.5 | 25.5 | 2083 | 41.9 | 58.1 |
|  | 2018 | 3009 | 73.5 | 26.5 | 2014 | 44.0 | 56.0 |
|  | 2019 | 3297 | 71.4 | 28.6 | 1916 | 41.1 | 58.9 |
|  | 2015-2019 | 4913 | 85.2 | 14.8 | 4428 | 62.0 | 38.0 |
| Specialist care | 2015 | 319 | 79.9 | 20.1 | 96 | 53.1 | 46.9 |
|  | 2016 | 421 | 77.2 | 22.8 | 110 | 57.3 | 42.7 |
|  | 2017 | 467 | 79.9 | 20.1 | 118 | 57.6 | 42.4 |
|  | 2018 | 517 | 77.6 | 22.4 | 135 | 48.1 | 51.9 |
|  | 2019 | 555 | 77.3 | 22.7 | 100 | 65.0 | 35.0 |
|  | 2015-2019 | 887 | 86.1 | 13.9 | 332 | 64.5 | 35.5 |
| Primary care | 2015 | 938 | 77.0 | 23.0 | 1669 | 44.4 | 55.6 |
|  | 2016 | 1239 | 76.5 | 23.5 | 1716 | 41.3 | 58.7 |
|  | 2017 | 1455 | 73.9 | 26.1 | 1711 | 40.7 | 59.3 |
|  | 2018 | 1708 | 74.0 | 26.0 | 1657 | 43.7 | 56.3 |
|  | 2019 | 1947 | 69.6 | 30.4 | 1623 | 39.5 | 60.5 |
|  | 2015-2019 | 2911 | 84.2 | 15.8 | 3528 | 61.5 | 38.5 |
| Shared care | 2015 | 524 | 74.2 | 25.8 | 314 | 43.9 | 56.1 |
|  | 2016 | 664 | 75.5 | 24.5 | 303 | 45.2 | 54.8 |
|  | 2017 | 695 | 72.1 | 27.9 | 254 | 42.5 | 57.5 |
|  | 2018 | 784 | 69.6 | 30.4 | 222 | 43.7 | 56.3 |
|  | 2019 | 795 | 71.7 | 28.3 | 193 | 42.5 | 57.5 |
|  | 2015-2019 | 1115 | 87.1 | 12.9 | 568 | 63.9 | 36.1 |
